# Temporal Anomalies in Immunological Gene Expression in a Time Series of Wild Mice: Signature of an Epidemic?

**DOI:** 10.1371/journal.pone.0020070

**Published:** 2011-05-23

**Authors:** Ida M. Friberg, Ann Lowe, Catriona Ralli, Janette E. Bradley, Joseph A. Jackson

**Affiliations:** 1 School of Biology, The University of Nottingham, Nottingham, United Kingdom; 2 IBERS, Aberystwyth University, Penglais, Aberystwyth, Ceredigion, United Kingdom; University of Edinburgh, United Kingdom

## Abstract

Although the ecological importance of coinfection is increasingly recognized, analyses of microbial pathogen dynamics in wildlife usually focus on an *ad hoc* subset of the species present due to technological limitations on detection. Here we demonstrate the use of expression profiles for immunological genes (pattern recognition receptors, cytokines and transcription factors) as a means to identify, without preconception, the likelihood of important acute microbial infections in wildlife. Using a wood mouse population in the UK as a model we identified significant temporal clusters of individuals with extreme expression of immunological mediators across multiple loci, typical of an acute microbial infection. These clusters were circumstantially associated with demographic perturbation in the summertime wood mouse population. Animals in one cluster also had significantly higher individual macroparasite burdens than contemporaries with “normal” expression patterns. If the extreme transcriptional profiles observed are induced by an infectious agent then this implicates macroparasites as a possible player in mediating individual susceptibility or resilience to infection. The form of survey described here, combined with next generation nucleic acids sequencing methods for the broad detection of microbial infectious agents in individuals with anomalous immunological transcriptional profiles, could be a powerful tool for revealing unrecognized, ecologically important infectious agents circulating in wildlife populations.

## Introduction

The role of infectious agents in the dynamics of natural vertebrate populations has been a growing interest for ecologists. Endemic infections have been shown to have important effects on survival [1], [2] and reproduction [3], [4], [5], [6], [7] and the theoretical potential to regulate populations and drive abundance cycles [8], [9]. At the same time there are many documented instances of epidemics associated with catastrophic mortality in nature [10], [11], [12], although the observation of such events may often be serendipitous or depend on the focus of the researcher. Increasingly it is also appreciated that co-occurring pathogens should be regarded as a potential community [13], [14], whose interactions may define the consequences of infection for the individual host and host population [15]. In general, however, ecological studies of microbial infectious disease in nature are constrained to the analysis of a single pathogen or an *ad hoc* panel of pathogens for which there is *a priori* knowledge, a preconception of possible importance and specific diagnostic reagents.

An alternative is to focus on non-pathogen-specific aspects of the host's immune response [16], detecting the presence of significant microbial infections through perturbations caused in the expression of immunological genes in the host [17]. In the present study we present an example of such an approach, revealing spatio-temporal patterns of immunological expression in a summertime wood mouse population in the UK that may point to epidemic infections with an unknown microbial infectious agent.

## Materials and Methods

### Study design

We sampled wood mice (*Apodemus sylvaticus*) on 12 occasions through May–December 2008 at Cotgrave Forest (+52.891, −1.041242) a ∼135 ha area of coniferous and mixed forestry plantation in Nottinghamshire, UK (total *n* = 141). Mice were caught by overnight live-trapping using Longworth traps. The traps were placed at permanently marked stations located at 20 m intervals (1 trap/station) along a 1600 m curvilinear transect that traced the margins of contiguous forestry blocks. These blocks were separated along their edges by narrow clear areas ∼10–40 m wide. The use of an extended trapping line was intended to distribute trapping effort over a very large area and avoid local demographic effects. Traps were set in the afternoon (16.00 h BST) and collected the following morning (08.00 h) throughout the study period. Females that were lactating or heavily pregnant at the time of capture were released. At the last two sampling points, where numbers of mice captured exceeded the capacity to process them, some randomly selected individuals were also released. Other individuals were processed and infection variables quantified as previously described [18]. This was with the exception that in the present study lice were recorded in different infection categories (absent, <50 egg cases present, ≥50 egg cases present). Additionally, in the present study, thin blood smears were prepared from cardiac blood and examined for the presence of *Bartonella* spp. and *Babesia microti* (counts of infected cells in one hundred 100× microscope fields). Eyes from each specimen were stored in 4% formalin and then lenses were removed and dried in an oven at 80°C for 72 h and weighed for use as an index of age (combined weight, g) [19]. In the present study the same set of macroparasites was found as in a previous study along the same transect in 2007 [18], with the exception that *Heterakis spumosa* was absent.

### Ethical statement

Use of animals in the present study complied with relevant UK legislation. Mice were killed by a listed competent person using a Home Office approved method as detailed in schedule 1 of the Animal (Scientific Procedures) Act 1986 (ASPA). In consultation with the Home Office ASPA inspectorate, the work was not considered to involve any scientific procedures on living animals (merely housing for a short period before killing [18]) and was therefore not subject to regulation by ASPA.

### Immunology

For each mouse we established five sets of splenocyte cultures. One of these was left unstimulated, to provide “constitutive” measurements and three others were respectively stimulated with agonists for toll-like receptor (TLR) 2, TLR4 and TLR9, which are receptors involved in the innate recognition of, and the triggering of immune responses against, invading microorganisms [20]. Cells and supernatants harvested from these cultures at 24 h were used to measure mRNA and protein expression of the pro-inflammatory cytokine tumour necrosis factor alpha (TNF-α) [18], [21], mRNA expression of TLR2, TLR4 and TLR9, and mRNA expression of TGF-β1 and IL-10, which are anti-inflammatory cytokines involved in the regulation of immune responses [22], [23], [24]. In the unstimulated cultures alone we measured mRNA expression of FoxP3, a transcription factor associated with the anti-inflammatory regulatory T-helper cell (T_reg_) subset [25], [26]. TNF-α protein expression was also measured in 96 h supernatants from a further set of cultures stimulated with *Heligmosomoides bakeri* somatic antigen (*HbAg*).

### Cell culture

Spleens were disaggregated through a 70 µm cell strainer into RPMI 1640. Following erythrocytic lysis (Sigma R7757), leucocytes were washed three times in RPMI 1640 and then cultured (37°C, 5% CO_2_) on 96 well plates at 2×10^6^ cells/ml in RPMI 1640 supplemented with 24 mM Na HCO_3_, 10% heat inactivated foetal calf serum, 2 mM L-glutamine, 100 u/ml penicillin, 100 µg/ml streptomycin and 60 µM monothioglycerol. Triplicate sets of individual cultures (150 µl volume) were assigned to each experimental condition. Three cultures sets were stimulated with different TLR agonists: heat-killed endotoxin-free *Listeria monocytogenes* (TLR2 agonist) at 0.6×10^8^ cells/ml, or a 50∶50 mix of Ultrapure *Escherichia coli* K12 lipopolysaccharide and Ultrapure *Salmonella minnesota* lipopolysaccharide (TLR4 agonists) at 3 µg/ml, or oligonucleotide ODN2006 (TLR9 agonist) at 6 µg/ml. All TLR agonist reagents utilized were tested for functional induction of the target TLR and for endotoxin contamination by the manufacturer (InvivoGen) and were used at concentrations previously determined to be optimal [18]. Further culture sets were stimulated with *HbAg* antigen at 50 µg/ml (pretreated with 500 U/ml polymyxin B for 4 h at 4°C) or left unstimulated. Culture supernatants and cells were harvested after 24 h for the unstimulated and TLR cultures and after 96 h for the *HbAg* cultures. Supernatants were immediately stored at −80°C and cells were placed overnight at 4°C in RNA*later®* (Ambion) and then stored at −80°C.

### TNF-α ELISA

Accumulated TNF-α protein was measured in 24 h culture supernatants by ELISA (R&D, DY410) as previously described [18], except that all samples were analysed in triplicate wells.

### Q-PCR

Relative mRNA accumulations in cells harvested at 24 h were measured by two-step reverse transcription quantitative real-time PCR (Q-PCR) using SYBR Green chemistry. Total RNA was extracted from cells stored in RNA*later®* using an Ambion RNAqueous®-Micro kit (AM1931) and converted to cDNA using an Applied Biosystems High capacity RNA-to-cDNA kit (4387406). All samples were DNase-treated and periodic controls lacking a reverse transcriptase enzymatic step indicated that DNA contamination was negligible. Real-time PCR primers for the cytokines, transcription factor and endogenous control genes were designed from conserved regions identified in multiple alignments of rodent sequences in the NCBI database. For TLR genes *de novo* amplification and sequencing of a larger (300–500 bp) fragment was first carried out using conserved primers (designed as above). Real-time PCR primers were then designed from the resulting *Apodemus sylvaticus* TLR sequence. All primers were designed with *Primer 3* v. 0.4.0. For each target and endogenous control gene 2–6 primer pairs were designed and trialled and the optimal set selected for further use. All selected primers (Table 1) were confirmed to be specific by re-sequencing of the target fragment, did not produce confounding primer dimers under assay conditions and had approximately equal efficiencies close to 100%, based on the average of 5-fold dilution series repeated on multiple different plates. Target genes were normalized against the endogenous control gene β-actin. This was the most stable endogenous control gene amongst a set of 9 candidates considered (including γ-actin, G6PDH, GAPDH, HPRT1, Tubulin, YWHAZ, 18 s and Albumin) as determined by stepwise exclusion of those with low stability estimated by the *M* statistic [27]. This procedure was conducted separately for 10 LPS-, HKLM- or ODN 2006-stimulated cultures randomly selected out of a pool of 300 culture samples. In all three cases β-actin was identified as the joint most stably expressed gene. The primers used for β-actin were sense 5′- AGATCAAGATCATTGCTCCTCCT -3′ and antisense 5′- TCCTGCTTGCTGATCCACATC -3′ which gave a 102 bp amplicon. The 96-well real-time PCR assay was designed so that all target genes for one animal were run on a single plate, except for Foxp3 amplifications which were all run on a separate plate. Each target gene reaction and the endogenous control gene reaction were run in duplicate for samples and in triplicate for a reference cDNA that was included on each plate. A no-template-control was included in duplicate for each primer pair. Real-time PCR assays were run using default settings and the manufacturer-recommended enzyme, buffer and SYBR Green reagents on an ABI 7500 Fast machine (Applied Biosystems). ABI 7500 v 2.0.4 software was used to calculate the expression of each target in each sample as a fold difference relative to the reference cDNA by the ΔΔCt method [28].

**Table 1:** Q-PCR primers used for the target immunological and TLR genes.

Gene	Primer sequence	Amplicon size (bp)	Molecule type	Function
Foxp3	*L* 5′- TAGGAGACATCCATCAGGGCTCTA	105	Transcription factor	Regulatory T-helper cells
	*R* 5′- AGCAACCTGGAGAAGATCTGTGA			
IL-10	*L* 5′- TTTAAGGGTTACTTGGGTTGC	109	Cytokine	Broad regulatory functions
	*R* 5′- TCAAATGCTCCTTGATTTCTG			
TGF-β1	*L* 5′- TCAGCTCCACAGAGAAGAACTGC	106		
	*R* 5′- AAGTTGGCATGGTAGCCCTTG			
TNF-α	*L* 5′- TCTACTGAACTTCGGGGTGATCG	119		Pro-inflammatory
	*R* 5′- GATGATCTGAGTGTGAGGGTCTGG			
TLR2	*L* 5′- TACTGGGTGGAGAACCTCATGG	101	Transmembrane receptor	Pattern recognition
	*R* 5′- ATGATCCATTTGCCCGGAAC			
TLR4	*L* 5′- TGACATCCCTTCTTCAGCCAAG	108		
	*R* 5′-GATAAATCCAGCCACTGAAGTTCTGA			
TLR9	*L* 5′- CCTGGCAGGCAACCAACTAA	110		
	*R* 5′- AGGCTGGGACCACAGAGACAAT			

### Data analysis

In total 30 immunological expression values were measured per individual. Initial examination of the data indicated a small number of individuals in which most or all of the readings were extremely high. In order to characterize this group each variable was log transformed (log10 [*x*+1]) and standardized ([*x*−mean]/standard deviation) and then outlying observations in each variable were determined as those occurring >1.5× the interquartile range above the third data quartile. Individual mice with ≥10 such outlying values were classified as part of an “extreme expression” group. Examining the subset of 22 variables in which there were few missing values and considering animals with a full set of observations for these, we also used hierarchical cluster analysis (HCA) (average link) and ordinations against the principal axes derived from principal co-ordinates analysis (PCO) and principal components analysis (PCA) to assess the distribution of individuals in phenotypic space. Data were log-transformed and standardized prior to HCA and PCO and log-transformed prior to PCA on the correlation matrix.

To analyze clustering in the occurrence of “extreme expression” status we coded extreme (1) *vs* non-extreme (0) expression as a binary variable and carried out retrospective spatial, temporal and spatio-temporal scans [29] with the software SaTScan™ v9.1.0, specifying a Bernoulli model. Following identification of a most significant cluster, subsequent clusters were identified by adjusting for the presence of more significant clusters.

Variations in the macroparasite community were summarised by a PCA of individual parasite variables on the correlation matrix [18]. This was based on data for the common macroparasite species at Cotgrave Forest [18]: *Skrjabinotaenia lobata*, *Brachylaemus recurvum*, *Calodium hepaticum*, *Syphacia nigeriana*, *Heligmosomoides polygyrus*, *Ixodes ricinus*, laelapid mites and *Polyplax serrata*. As the PCA of macroparasite variables produced a significant (*P*<0.001) first component with coefficients of the same sign for most species, scores for this component (PC1*^P^*) were taken as an overall indicator of macroparasite infection.

An estimator of body condition was produced by taking residuals (BW*^resid^*) from a regression of body weight (BW) on SVL, incorporating different slopes for males and females and quadratic terms to allow for a curvilinear association.

We used variations in trapping catch rate as a measure of demographic fluctuation in the host population as this rate would be expected to reflect either changes in population density or changes in spatial behaviour that relate, in some way, to the condition of individuals. Initial pairwise comparisons between trap success on different sampling occasions were based on contingency table analysis (Fisher exact tests). Overall variation in capture rates was then analysed by a linear mixed model (LMM) allowing for correlation between successive trapping intervals (AR1 covariance model).

Variations in body condition and PC1*^P^*were assessed with general linear models (LMs) initially including terms for cluster membership, body size (SVL) and host stage (classified as in Jackson *et al.* [18]), with deletion of non-significant terms. For analyses of individual macroparasite species, LMs with normal errors or equivalent generalized linear models (GLMs) with Poisson errors or negative binomial errors were used as appropriate (again with deletion of non-significant terms). A Poisson distribution was used as a surrogate distribution in the case of ordinal data for *P. serrata*. In the case of negative binomial errors, the dispersion parameter *k* was estimated from that of the entire host population and any significant results reported are also consistent with an analysis of log-transformed data in an LM.

## Results

### Individual variation in immunological expression: evidence of acute infection

**Figure 1:**
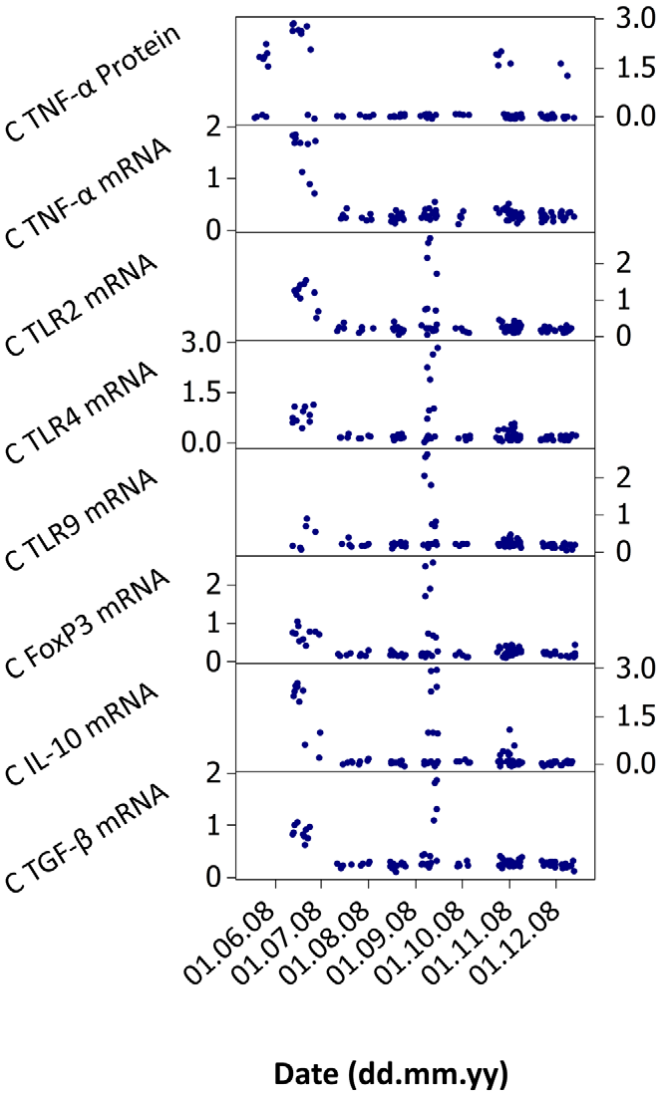
Patterns of immunological expression in an *Apodemus sylvaticus* population in the UK across May–December 2008. Scatter of relative expression data for individuals are shown on a log scale (log_10_ (*x*+1)) for all constitutive (C) measurements taken in unstimulated splenocyte cultures. Many animals with anomalous expression profiles were noted in June and September 2008. Similar temporal patterns emerged for cultures subject to stimulatory conditions (not shown).

**Figure 2:**
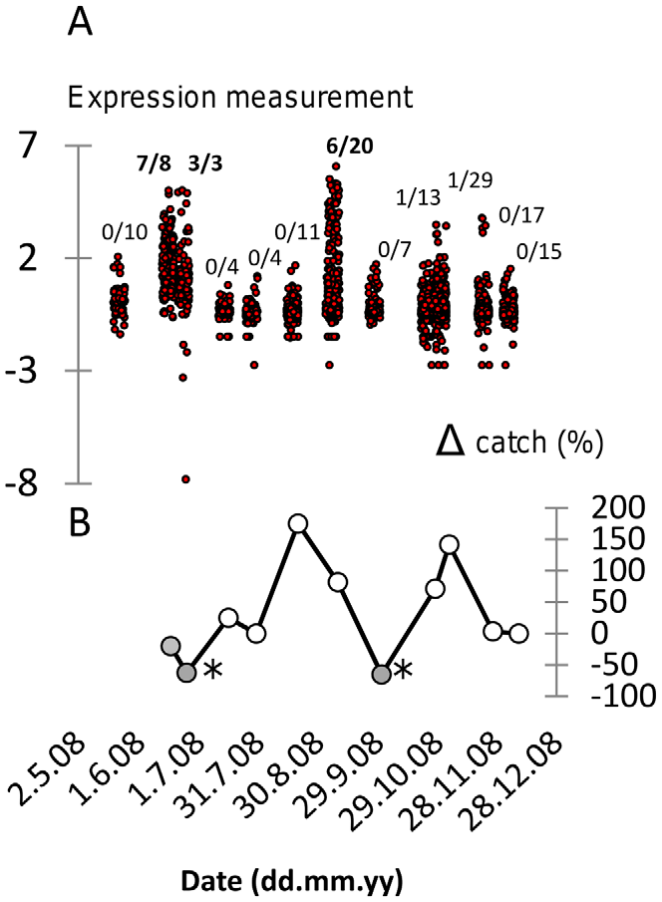
Association between extreme immunological gene expression and live-trapping catch rate in an *Apodemus sylvaticus* population in the UK across May–December 2008 (12 temporal sampling points). A. Superimposed plot of all immunological gene expression measurements showing spikes of extreme activity in late June and early September (variables log-transformed and standardized, [*x*−mean]/standard deviation). Fractions show proportion of individuals with expression profiles classified as extreme at each sampling point. Fractions shown in bold indicate the contribution of individuals with extreme immunological expression at the corresponding sampling point to a statistically significant (*P* = 0.001) temporal cluster. B. Percentage change in catch rate (Δ catch) between sampling points. There was a highly significant overall positive trend in catch rate with time (*P*<0.001) but closed circles indicate instances of negative growth in catch rate (*, catch rate significantly less than at 1 sampling point or 2 sampling points earlier).

**Figure 3:**
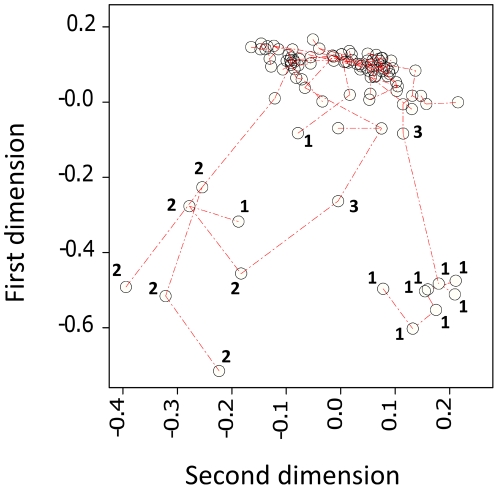
Minimum spanning tree, corresponding to a hierarchical cluster analysis, projected onto the scatter of individual mice against the two major dimensions from a principal coordinates analysis of 22 immunological expression variables. Labelled data points relate to individuals classified as showing extreme immunological expression on the basis of ≥10 outlying expression measurements. Individuals are further identified as belonging to a significant temporal cluster in June (1) or a significant temporal cluster in September (2). Individuals labelled 3 are unassigned to a temporal cluster. Individuals from cluster 1 and cluster 2 were mostly well differentiated from each other and the main series of individuals, although a few atypical individuals from cluster 1 were intermediate or more similar to individuals in cluster 2. This interpretation is consistent with outputs from principal components analysis (not shown).

We analysed splenocyte expression of immunological genes (TLR2, TLR4, TLR9, TNF-α, TGF-β1, IL-10, FoxP3) in a time series of *A. sylvaticus* at Cotgrave Forest, UK from May to December 2008. Preliminary analyses of individual variation indicated that a small proportion of individuals were associated with very extreme expression values for most or all of the measurements taken (Figure 1). In order to categorize this variation we initially identified outlier values in log-transformed, standardized data as occurring above the third data quartile cut-off by more than 1.5× the interquartile range. Individuals with >10 outlier readings from the 30 variables measured were considered as an “extreme expression” group (Figure 2). The individual composition of this group was broadly supported by HCA, PCA and PCO for the subset of 22 expression variables without missing data values (Figure 3).

It was not possible to identify a candidate infectious aetiological agent for the extreme immunological expression syndrome described above amongst the microparasites (*Eimeria* spp., *Babesia microti* and *Bartonella* spp.) and macroparasites whose presence we recorded. These are all endemic at Cotgrave Forest (Jackson *et al.* 2009; unpublished data) and frequently occur in hosts with non-extreme levels of immunological expression.

### Temporal clustering

We categorized individuals into “extreme” and “normal” expression groups (as above) and scanned for temporal clusters of extreme expression using a Bernoulli model in the programme SaTScan™. A significant temporal cluster (cluster 1) was detected in late June 2008 (log likelihood ratio = 20.37, *P* = 0.001). Adjusting for the occurrence of the most significant cluster, a significant secondary temporal cluster (cluster 2) occurred in early September 2008 (log likelihood ratio = 13.10, *P* = 0.001). Analyses for spatial clusters and spatial-temporal clusters failed to detect any purely spatial clustering, but did find two significant space-time clusters (*P* = 0.014) within the time frame of the most significant temporal cluster. The areas included and not included in these clusters appeared to broadly correspond to distinct forestry blocks.

### Temporal relationship of clusters to demographic variation

Against a background of apparent demographic increase during May–December 2008, significant decreases in trapping rate with respect the immediately preceding trapping occasions (either 1 or 2 intervals distant) were only recorded immediately after the detection of extreme expression clusters (Figure 2). LMM analysis of trapping success data against time (days), allowing for positive autocorrelation between successive trapping sessions, suggested a highly significant increasing trend across May to December (LMM, *P*<0.001; parameter estimate = 0.00130±0.00035 catch rate day^−1^). A 2-level factor coding the two time intervals following the first detection of each extreme expression cluster (*vs* other time intervals) was also significant (*P*∼0.001; parameter estimate = −0.117±0.036).

**Figure 4:**
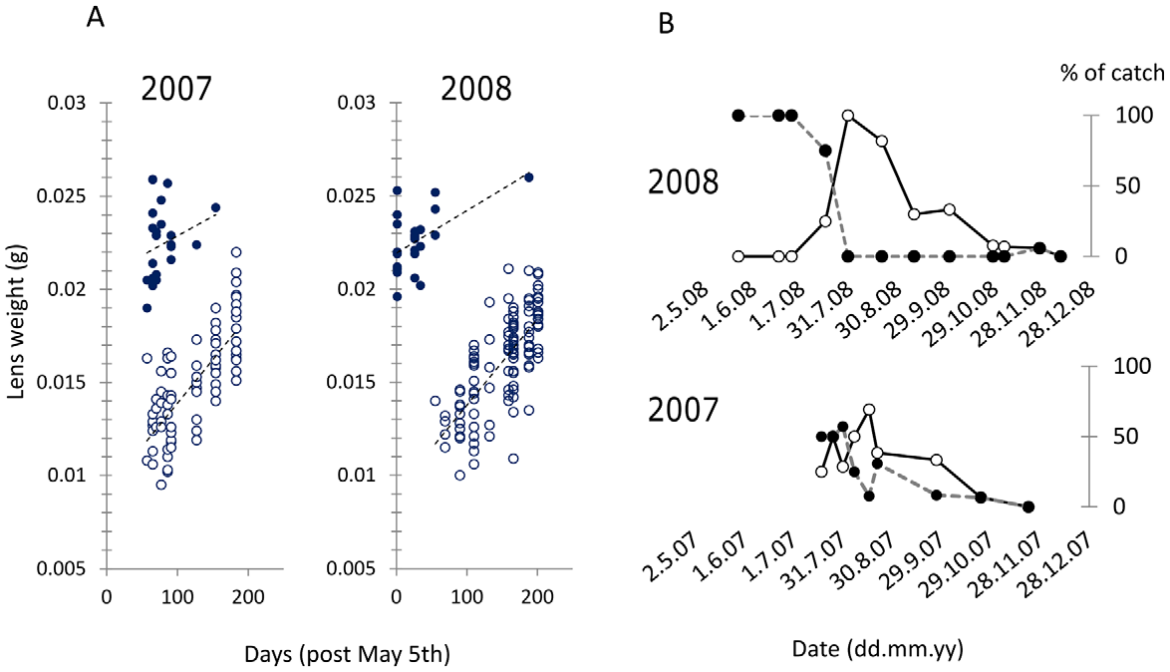
Turnover of *Apodemus sylvaticus* year cohorts at Cotgrave Forest in 2007 and 2008. A. Year cohorts could be unambiguously classified in 2007 and 2008 by plotting combined dry eye lens weight against date of capture. Closed circles indicate over-wintered (1^+^) animals; open circles indicate young-of-the-year (0^+^). Least squares regression lines shown for reference. B. Contrasting cohort dynamics in 2007 and 2008. Closed circles indicate over-wintered (1^+^) animals; open circles indicate the most recent young-of-the-year (0^+^) recruits with eye lenses <0.014 g. In 2008 1^+^ animals predominantly died off in June, coincident with a large pulse of recruitment. In 2007 1^+^ animals persist in significant numbers into August and new recruits never make up the entire population. Data for 2007 based on retrospective analysis of *A. sylvaticus* sampled by Jackson *et al.* [18].

Using eye lens weight as an index of age, plots of lens weight with respect to time allowed unambiguous identification of over-wintered (1^+^) and young-of-the-year (0^+^) cohorts in 2008 and also in a retrospective analysis of animals sampled at Cotgrave Forest by Jackson *et al.* [18] in 2007 (Figure 4). Cohort dynamics differed in 2008 compared to 2007 by an abrupt earlier disappearance of 1^+^ animals, in late June, that was followed by a very large proportional contribution to the population by new recruits (0^+^ individuals with the lightest eye lenses, <0.014 g) (Figure 4).

### Between cluster variation in expression

**Figure 5:**
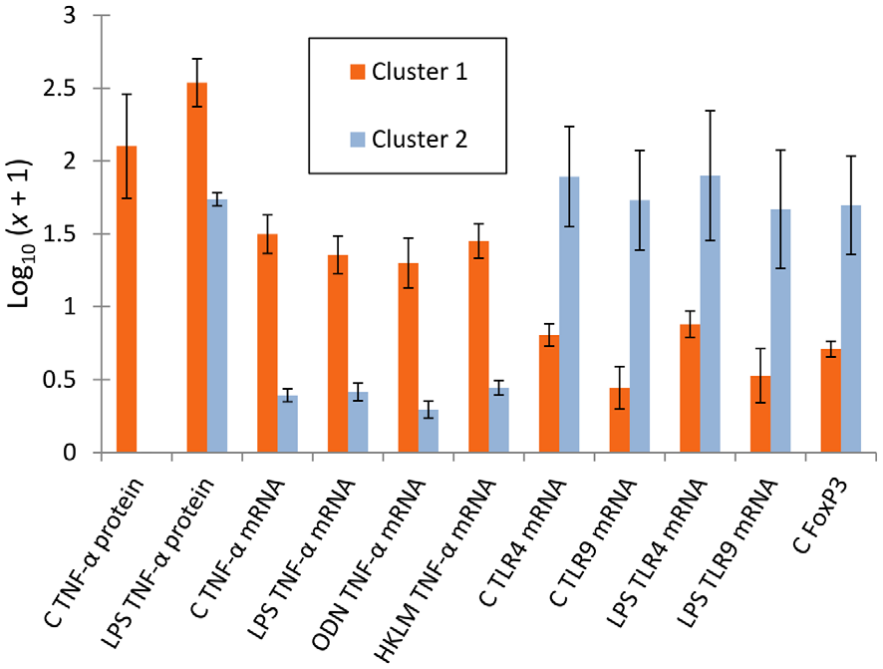
Contrasting immunological gene expression profiles in animals from different significant temporal clusters of extremely high immunological expression. Bars show mean expression in variables where a pair-wise significant difference occurred (*P*<0.05). Cluster 1 individuals (June) were characterized by higher expression of the pro-inflammatory cytokineTNF-α. Cluster 2 animals (September) were characterized by higher expression of the toll-like receptors, TLR 4 and TLR9 and the regulatory T-helper cell transcription factor FoxP3. (C, measurements in unstimulated 24 h splenocyte cultures; HKLM, measurements in 24 h splenocyte cultures stimulated with HKLM; LPS, measurements in 24 h splenocyte cultures stimulated with LPS; ODN, measurements in 24 h splenocyte cultures stimulated with ODN 2006.)

Although a small number of divergent individuals occurred within cluster 1 (Figure 3), significant overall variation in expression patterns occurred between cluster 1 and cluster 2 (Figure 5). Most individuals in cluster 1 could be characterized by comparatively very elevated constitutive and LPS-stimulated TNF-α expression. Individuals in cluster 2 were characterized by relatively high levels of constitutive and LPS-stimulated expression of TLR4, TLR9 and FoxP3.

### Associations of cluster 2 with individual condition and macroparasite infection

**Figure 6:**
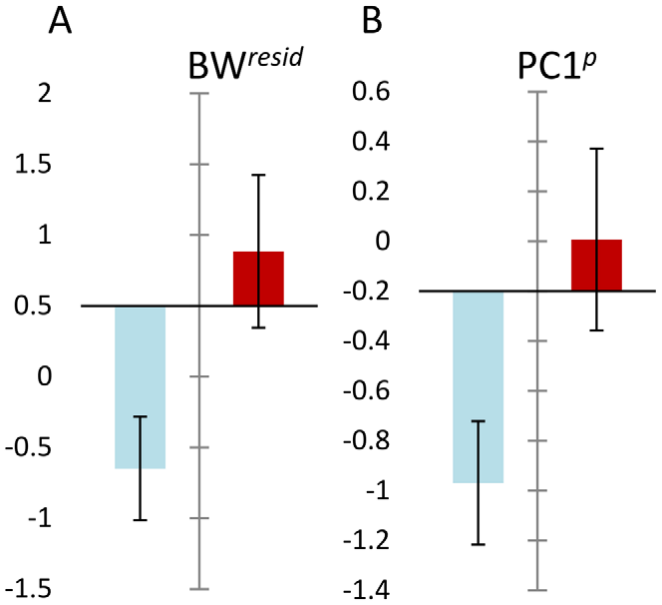
Differences in body condition and macroparasite burden between cluster 2 and non-cluster animals captured at the same time. A. Cluster 2 animals (dark bar) had significantly better body condition (BW*^resid^*) than non-cluster animals, adjusting for size and stage. B. Cluster 2 animals (dark bar) had greater grouped macroparasite burdens (PC1*^P^*) than non-cluster animals, adjusting for size and stage. PC1*^P^* represents first principal component scores from a principal components analysis of common macroparasite species at Cotgrave Forest. This principal component accounted for 23% of total variation and showed high loadings of the same sign for most species. It was thus taken to represent the major pattern of positive covariation within the assemblage. Variable coefficients on PC1*^P^* were: *Skrjabinotaenia lobata* 0.25, *Brachylaemus recurvum* 0.28, *Calodium hepaticum* 0.24, *Syphacia nigeriana* 0.52, *Heligmosomoides polygyrus* 0.50, *Ixodes ricinus* −0.040, laelapid mites 0.102, *Polyplax serrata* 0.52. There were individually significant associations with cluster 2 membership, in the same direction as PC1*^P^*, for *P. serrata* and *S. lobata*, adjusting for host size and stage.

A comparison of contemporaneous cluster 1 and non-cluster animals was not possible (insufficient individuals occurred in the same time intervals) but cluster 2 individuals had significantly greater body condition (BW*^resid^*) (LM, *P* = 0.036) and grouped macroparasite infection (PC1*^p^*) (LM, *P* = 0.048) than non-cluster individuals from the same sampling points (Figure 6). For individual macroparasite species, there were significantly greater infections in cluster 2 animals for the louse, *P. serrata* (GLM, Poisson errors, *P* = 0.048) and the catenotaeniid cestode, *S. lobata* (GLM, negative binomial errors, *P* = 0.035).

## Discussion

The analysis of immunological expression profiles may provide an unbiased strategy for identifying important microbial infection pressures acting on a natural population. In this study we attempted to demonstrate such an approach using a time series of wood mice from a habitat in the east midlands, UK.

Acute infections with highly pathogenic viruses, bacteria or protozoa are often associated with extreme expression of cytokines and TLRs [30], [31], [32], [33]. We discovered significant temporal clusters of individuals with extreme expression of immunologically-relevant genes, including cytokines and TLRs, in late June (cluster 1) and early September 2008 (cluster 2). This is consistent with the presence of an acute, highly immunogenic microbial infection in the mouse population at these times. Other possible causes for the “extreme expression” clusters are considered less likely. Thus, autoimmunity, allergy or malignancy not involving infection does not well explain the clustered temporal epidemiological pattern and poisoning is unlikely because the habitat is managed for pheasant shooting (pheasants would be likely to take poisoned bait placed for rodents) . The pattern seen is also difficult to attribute to handling stress or to ontogenetic or genetic variation. Compared to the non-cluster animals in the present study and to hundreds of other “normal” wild rodents (*A. sylvaticus* and *Microtus agrestis*) recently analysed in our laboratory in a similar way [16], [18], the relative mRNA expression ratios for immunological genes and the amount of constitutive TNF- *α* protein secretion in some cluster animals are unprecedentedly high. The variation in expression between cluster and non-cluster animals also seems to be much greater than constitutive variation in expression reported amongst wild-derived *Mus* inbred laboratory lines [34], [35].

The occurrence of the clusters in time was circumstantially associated with significant reductions in *A. sylvaticus* trapping rate that were contrary to a prevailing trend of demographic increase during the period of study. An additional, circumstantial, corollary was that an abrupt turnover between 1^+^ and 0^+^ *A. sylvaticus* cohorts occurred in the weeks following cluster 1, consistent with a perturbation affecting the population at this time. Over an equivalent time interval in the preceding year (2007), clusters of individuals with extreme TNF-α protein expression did not occur ([18]; unpublished data) and the turnover between 1^+^ and 0^+^ cohorts proceeded much more gradually (Figure 4).

Whilst the identity of any putative aetiological pathogen(s) is unknown, the two “epidemic” clusters differed in the nature of the immune responses observed. Members of both clusters expressed high levels of all immunological genes studied, but cluster 1 was characterized by relatively higher expression of TNF-α at the protein and mRNA level and cluster 2 by higher expression of TLR4 and TLR9 and the regulatory T-helper cell-associated transcription factor FoxP3. These differences may be indicative of more than one infectious agent stimulating different immune responses. Alternatively, different immune responses to the same pathogen may have occurred because the animals involved in the respective clusters were themselves different. Cluster 1 individuals were exclusively over-wintered (1^+^) animals, whilst cluster 2 animals were exclusively young-of-the-year (0^+^) individuals. Phenotypic differences might therefore have arisen from ontogenetic stage variation, or from a different history of programming by environmental cues in the respective cohorts. A further possibility is that animals in the second cluster might have had some level of acquired immunity, following the outbreak earlier in the year, and produced more highly regulated responses indicative of a degree of immunological tolerance. This latter possibility is supported by the fact that some atypical individuals occurred in cluster 1 that were either phenotypically intermediate, or, more similar to cluster 2 individuals (Figure 3).

Given the highly focussed temporal occurrence of the clusters (appearing and disappearing over the timescale of a few weeks), it might be inferred that if a putative infectious agent is involved this is highly transmissible and produces acute infections that rapidly result in death or recovery and the development of resistance. It is likely such a pathogen could only be maintained by rapid spatial movement through the wider population, via locally unstable dynamics, or by inactive latent infections in some hosts acting as a reservoir and intermittent source of infection through time. One possibility consistent with some of these considerations is cowpox virus, infections with which can show fine-scale temporal clustering in wood mouse populations [36]. However, cowpox virus is known to suppress TNF-α responses [37], which were expressed at very high levels in cluster 1 mice. Furthermore, field studies of cowpox in UK wild rodents [38], [39] generally indicate that infection levels within populations persist with a much coarser temporal grain than was the case for the tightly focussed episodes of infection recorded here.

A further interesting characteristic of the second cluster is that cluster members, controlling for life history stage and size, showed significantly better body condition and greater macroparasite infection than non-cluster individuals from the same time interval. This indicates that healthier individuals with more macroparasites might either be more susceptible to any putative infection or more resilient to the effects of infection. Increased susceptibility could arise because individuals in better condition eat more and range farther, exposing themselves to greater infection risks from macroparasites and microbial infections. Macroparasites might also contribute to susceptibility due to their anti-inflammatory activities [18]. Increased resilience could occur because animals in better starting condition might be more likely to survive infection, thus raising their representation in infected classes by differential mortality. The immunoregulatory influence of macroparasites might also be important in this context: dampening potentially lethal immunopathogenic inflammatory responses. A further possibility is that individual mice respond to infection by adjusting life history traits in a way that produces an overcompensatory response in body condition. For example, *A. sylvaticus* infected with cowpox virus in summer may show positive effects on survival due to a diversion of resources from reproduction [3].

We have shown here that very distinct anomalies in immunological gene expression patterns can be detected in wild mammal populations if these are surveyed over time. We suggest that such patterns will often be caused by infectious agents and can be used to monitor important infection influences operating on a natural host population. The general approach used here is technically accessible to a wide range of researchers. An obvious extension would also be to combine the present methods with next generation sequencing techniques for the broad detection of microbial pathogens [40], [41] in animals with anomalous immunological transcriptional profiles compared to “normal” animals. In the present example, we focussed on a population of wood mice and were able to detect the probable signatures of epidemic infectious disease outbreaks and their demographic and individual correlates. Given the long standing interest in the dynamics of *A. sylvaticus* populations in the UK [42], [43], [44], [45], in particular the apparent slow growth during the earlier part of the breeding season, this study might point towards a possible, destabilizing, role for microbial infectious disease in addition to the factors identified by other authors. There is also a wider significance for the ability of ecologists to analyze the dynamic interaction between pathogen communities and their wildlife hosts and, particularly, to identify unsuspected acute microbial infectious disease outbreaks, whose effects might otherwise be dismissed as generalized environmental noise.
